# Genome-wide identification of bHLH transcription factors and functional analysis in salt gland development of the recretohalophyte sea lavender (*Limonium bicolor*)

**DOI:** 10.1093/hr/uhae036

**Published:** 2024-02-02

**Authors:** Xi Wang, Baoshan Wang, Fang Yuan

**Affiliations:** Shandong Provincial Key Laboratory of Plant Stress, College of Life Sciences, Shandong Normal University, Ji’nan, Shandong 250014, China; Shandong Provincial Key Laboratory of Plant Stress, College of Life Sciences, Shandong Normal University, Ji’nan, Shandong 250014, China; Shandong Provincial Key Laboratory of Plant Stress, College of Life Sciences, Shandong Normal University, Ji’nan, Shandong 250014, China

## Abstract

Transcription factors with basic helix–loop–helix (bHLH) structures regulate plant growth, epidermal structure development, metabolic processes, and responses to stress extensively. Sea lavender (*Limonium bicolor*) is a recretohalophyte with unique salt glands in the epidermis that make it highly resistant to salt stress, contributing to the improvement of saline lands. However, the features of the bHLH transcription factor family in *L. bicolor* are largely unknown. Here, we systematically analyzed the characteristics, localization, and phylogenetic relationships of 187 identified bHLH family genes throughout the *L. bicolor* genome, as well as their *cis*-regulatory promoter elements, expression patterns, and key roles in salt gland development or salt tolerance by genetic analysis. Nine verified *L. bicolor* bHLH genes are expressed and the encoded proteins function in the nucleus, among which the proteins encoded by *Lb2G14060* and *Lb1G07934* also localize to salt glands. Analysis of CRISPR-Cas9-generated knockout mutants and overexpression lines indicated that the protein encoded by *Lb1G07934* is involved in the formation of salt glands, salt secretion, and salt resistance, indicating that bHLH genes strongly influence epidermal structure development and stress responses. The current study lays the foundation for further investigation of the effects and functional mechanisms of bHLH genes in *L. bicolor* and paves the way for selecting salt-tolerance genes that will enhance salt resistance in crops and for the improvement of saline soils.

## Introduction

bHLHs are ubiquitous proteins in eukaryotes characterized by the occurrence of conserved basic helix–loop–helix (bHLH) motifs. In plants the bHLH domain, comprising nearly 60 amino acids [1, 2], plays a bridging role by binding to the promoter sequences of downstream genes, thus promoting development, growth, metabolic regulation, and tolerance of environmental stress [3]. The bHLH-type genes form one of the largest superfamilies in plants: 162 bHLH genes have been certificated in the model organism *Arabidopsis thaliana* [4], 122 in pepper [5], 124 in potato [6], 142 in cucumber [7], 155 in common bean [8], and 460 in *Brassica napus* [9].

It is generally believed that bHLH genes are involved in signal transduction and metabolic regulation in plants, including anthocyanin biosynthesis, tryptophan biosynthesis, gibberellin biosynthesis, and light signal transduction [7, 10, 11], as well as plant resistance to abiotic stress, such as extreme temperatures, drought, and salt stress. In the peanut (*Arachis hypogaea*) genome, one study identified 261 bHLH transcription factors and evaluated their functions in pod development [12]. Another study identified 137 bHLH genes in Jilin ginseng [13], some of which participate in plant responses to saline stress. In Chinese white pear (*Pyrus bretschneideri*), 197 bHLH genes were identified, most of which play vital roles in drought and cold tolerance [14]. Moreover, authentication of the bHLH genes of several other Rosaceae species uncovered 112, 129, 188, and 122 genes in strawberry (*Fragaria vesca L.*), peach (*Prunus persica* (L.) Batsch), apple (*Malus pumila* Mill.) and Chinese plum (*Prunus mume* Siebold & Zucc.), respectively, many of which have marked effects on stress responses [15]. Many bHLH genes from wheat (*Triticum aestivum*), rice (*Oryza sativa*) and maize (*Zea mays*) participate in plant abiotic stress [16].

Globally, more than 800 million hectares of cultivated land are currently harmed by high salinity [17]. It is estimated that soil salinization will impinge on over 20% of global irrigated farmland by 2050 [18, 19], seriously threatening food production and food security [20]. Enough arable land must be maintained to produce sufficient food to feed the worldwide population. Finding approaches to make use of saline alkaline land requires effective methods to improve crop salt tolerance. Mining salt-tolerance genes from halophytes may inform approaches to the improvement of salt resistance.

Sea lavender (*Limonium bicolor*) is a dicotyledonous recretohalophyte belonging to the genus *Limonium* in the family Plumbaginaceae, which can complete its lifecycle in saline soil (≥200 mM NaCl) [21], making it highly suitable for transforming saline soil. The leaves of *L. bicolor* contain typical salt-secreting structures in the epidermis, known as salt glands, that secrete excess salt to allow these plant to thrive in adverse environments [22]. The salt gland of *L. bicolor* is composed of 16 cells in an actinomorphic pattern of four secretory cells, four accessory cells, four inner cup cells and four outer cup cells. In order to investigate in depth the salt gland differentiation pattern, the first true leaf of *L. bicolor* was tracked, and was divided into five stages, comprising the undifferentiated stage (stage A), salt gland differentiation stage (stage B), stomatal differentiation stage (stage C), epidermal cell differentiation stage (stage D), and mature stage (stage E) [23]. These findings indicated that the salt gland was a completely different epidermal structure from stomata, and differentiated earlier than stomata.

Salt glands showed the characteristics of the autofluorescence phenomenon with four typical autofluorescent foci under 330–380 nm ultraviolet excitation [23]. The transcriptome of *L. bicolor* was measured and corresponding background databases were constructed [23, 24]. The establishment of the transformation and regeneration system [25] provided technical support to obtain overexpression and knockout lines of *L. bicolor*, which lay the foundations for research on salt gland development and salt secretion-related genes [26–28]. Recently the whole-genome sequencing and assembly of *L. bicolor* have been completed (BioProject number PRJNA753199), which laid a solid foundation and is an important source for the identification of the gene family [28].

The bHLH transcription factor encoded by *Lb1G04899* (*LbbHLH*) has important effects on salt gland differentiation and salt resistance in *L. bicolor* [28, 29]. However, no other bHLH-type genes have been studied in this species. Given that bHLH-type genes contribute to plant development and stress responses extensively, investigating this family seemed a fruitful approach to reveal their possible roles in salt tolerance. Here, we identified and characterized the bHLH transcription factor genes and proteins in *L. bicolor*, including their chromosomal locations, evolutionary relationships, conserved domains, *cis* elements, expression patterns, and subcellular localizations. Our findings lay a solid foundation for further exploration of the effects of bHLH-type genes in promoting salt gland formation and enhancing stress resistances in *L. bicolor*.

## Results

### Comprehensive identification and analysis of the *L. bicolor* bHLH family

**Figure 1:**
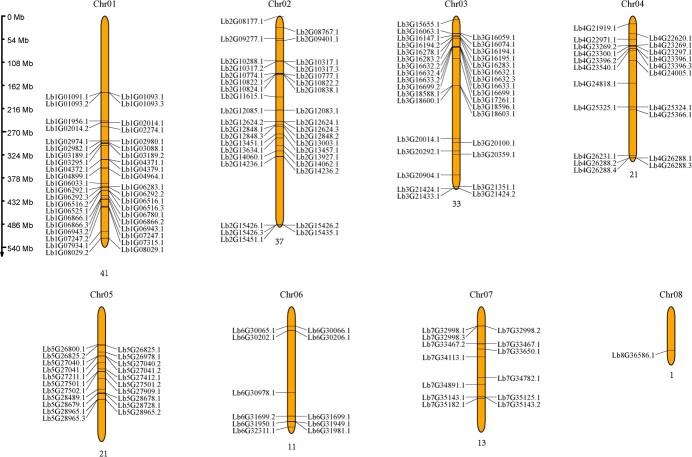
Chromosomal locations of 178 LbbHLH family genes in *L. bicolor*. The chromosome number is displayed above each chromosome. Chromosome length is expressed in Mb.

We identified 187 bHLH family genes in the genome of *L. bicolor*. The appearance of bHLH-type domains in these genes was confirmed using the Pfam_scan program. The 187 bHLH proteins are predicted to range from 94 (encoded by *Lb7G34891*) to 1548 (encoded by *Lb5G28679*) amino acids long, with MWs between 10.8323 kDa (encoded by *Lb7G34891*) and 171.14 kDa (encoded by *Lb5G28679*) based on their amino acid sequences. The pIs are distributed between 4.32 (encoded by *Lb4G23396*) and 10.84 (encoded by *Lb1G07934*) (Supplementary Data Table S1). Analysis based on previous genome sequencing data [28] revealed that 178 bHLH genes are found on the eight chromosomes of *L. bicolor* (Fig. 1), including 41 genes on Chr1, 37 genes on Chr2, 33 genes on Chr3, 21 genes each on Chr4 and Chr5, 11 genes on Chr6, 13 genes on Chr7, and only 1 gene on Chr8. The nine remaining genes are distributed on scaffolds (fragments not attached to chromosomes).

After identifying bHLH members in *Arabidopsis* using the same method as for *L. bicolor*, we merged the protein sequences of the bHLH members in *L. bicolor* and *Arabidopsis* and used them as input in the MUSCLE program for global sequence alignment. We used the alignment results to build a maximum-likelihood tree with the MEGA program, and finally optimized the results using iTOL. The proteins were divided into 11 subfamilies (Supplementary Data Fig. S1), each of which contained both AtbHLHs and LbbHLHs. To date, many studies have analyzed the functions of bHLH genes in *Arabidopsis*, but few have focused on these genes in *L. bicolor*. Therefore, the homology between LbbHLHs and AtbHLHs prompted us to carry out in-depth research on these genes in *L. bicolor*.

### Intraspecific and interspecific collinearity analysis

**Figure 2:**
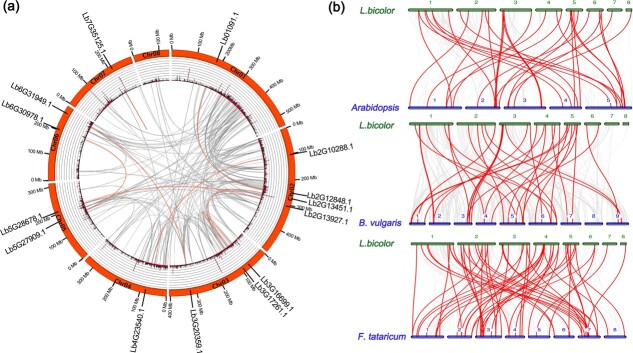
Intraspecific and interspecific collinearity analysis of bHLH genes. **a** Interchromosomal relationships of LbbHLH genes shown in a schematic. Gray lines indicate collinear blocks and red lines indicate bHLH family members in collinear blocks. **b** Interspecific analysis of bHLH genes between *Arabidopsis*, *B. vulgaris*, and *F. tataricum* and *L. bicolor*. Red lines indicate collinear blocks of bHLH genes within the *Arabidopsis*, *B. vulgaris*, *F. tataricum*, and *L. bicolor* genomes.

We identified collinear blocks in the *L. bicolor* genome using the MCScanX program, extracted the bHLH family members in these blocks, and used the Circos program to display the results (Fig. 2a). The *L. bicolor* genome contains six bHLH gene pairs, which are located on different chromosomes, indicating that the enlargement of the bHLH family likely depended on the replication of fragments in these regions. To better explore the evolution of the bHLH family, we constructed three comparative allograms of *L. bicolor* at the genome level. We selected three dicotyledons for analysis: *Arabidopsis*, *Beta vulgaris*, and *Fagopyrum tataricum*. *Beta vulgaris* and *F. tataricum* share high phylogenetic and evolutionary similarity with *L. bicolor* [28], and *Arabidopsis* is an important model organism. After identifying bHLH family members in all three species, we extracted collinear blocks between *L. bicolor* and *Arabidopsis*, *B. vulgaris*, and *F. tataricum* via JCVI, selected bHLH family members in these collinear blocks, and constructed a drawing using the JCVI drawing subroutine. The greatest number of bHLH homologs are present in *F. tataricum*, followed by *Arabidopsis* and *B. vulgaris* (Fig. 2b). Moreover, both *L. bicolor* and *F. tataricum* have eight chromosomes. Therefore, *F. tataricum* has the closest relationship with *L. bicolor* among the three species investigated.

### Analysis of gene structures and *cis* elements in the bHLH gene promoters

Eight conserved motifs were identified in the *L. bicolor* bHLH genes. Most of these genes contain introns, exons (yellow ellipses in Supplementary Data Fig. S2) and HLH domains (red rectangles); some also contain bHLH-MYC_ N motifs (blue rectangles). Most of these genes are distributed on Chr1–Chr4, Chr6, and Chr7, and none of those on Chr5 and Chr8 contain bHLH-MYC_ N motifs (Supplementary Data Fig. S2). Four motifs, Retrotran_ gag_ 2 (dark green rectangles), gag_ pre-integers (gray rectangles), RVT_ 2 (light red rectangles), and rve (yellow rectangles), were detected only in *Lb5G28679.1*. In addition, *Lb7G34782.1* contains the NAD_ binding_4 motif (bright green rectangle), which is unique to this gene.

We examined the promoter part of each bHLH gene using the bedtools program and predicted the *cis*-regulatory elements in these promoter regions using PlantCARE (Supplementary Data Table S2). Many stress-related elements exist in the promoter portions of the bHLH genes, such as elements related to xenobiotic stress, disease resistance, and responses to low temperature and drought (Supplementary Data Fig. S3). We also identified many phytohormone response elements, including elements related to auxin, salicylic acid, gibberellin, and abscisic acid, and many *cis*-acting elements involved in plant epidermal structure development and growth, such as elements conferring gene expression in the root, leaf, shoot, and seed. Thus, a variety of *cis* promoter elements regulate bHLH gene expression, including those related to the benign growth of salt glands and stress responses of *L. bicolor* possibly.

### Expression status of bHLH genes in *L. bicolor*

We calculated the expression levels of 187 bHLH genes based on the transcriptome data during specific developmental stages and time points after NaCl treatment and generated hierarchical clustering heat maps using the pearmap program in R. Almost half of the 187 family bHLH members, such as *Lb7G34891*, were expressed highly during the undifferentiated stage of salt gland development (represented by red rectangles; Supplementary Data Fig. S4a). The high expression levels of these genes prior to salt gland differentiation likely paves the way for the formation of salt glands. In other words, these genes maybe exerting significant influence in the differentiation and formation of salt glands. Some bHLH genes were highly expressed shortly after NaCl treatment (within 12 h), while others were highly expressed long after NaCl treatment (24–72 h; Supplementary Data Fig. S4b). The expression of the 187 genes thus did not exhibit a consistent pattern, suggesting that not all of them may be sensitive to NaCl treatment.

### Subcellular localization and *in situ* hybridization

In order to validate the expression specificity of bHLH family genes, nine representative genes were selected, distributed in eight different chromosomes. In particular, *Lb7G34891.1* was chosen due to the minimum number of encoded amino acids, while *Lb5G28679.1* encoded the maximum number. Besides, *Lb1G07934.1* had maximum PIs among all bHLH genes, and *Lb7G34782.1* had a unique NAD_binding_4 motif. In addition, another five bHLH genes were also selected on the other four chromosomes, including *Lb2G14060.1* on chromosome 2, *Lb3G16699.1* on chromosome 3, *Lb4G24818.1* on chromosome 4, *Lb6G30066.1* on chromosome 6, and *Lb8G36586.1* on chromosome 8. This screening criterion can ensure the verification of at least one gene on each chromosome, and also take genetic characteristics into account.

**Figure 3:**
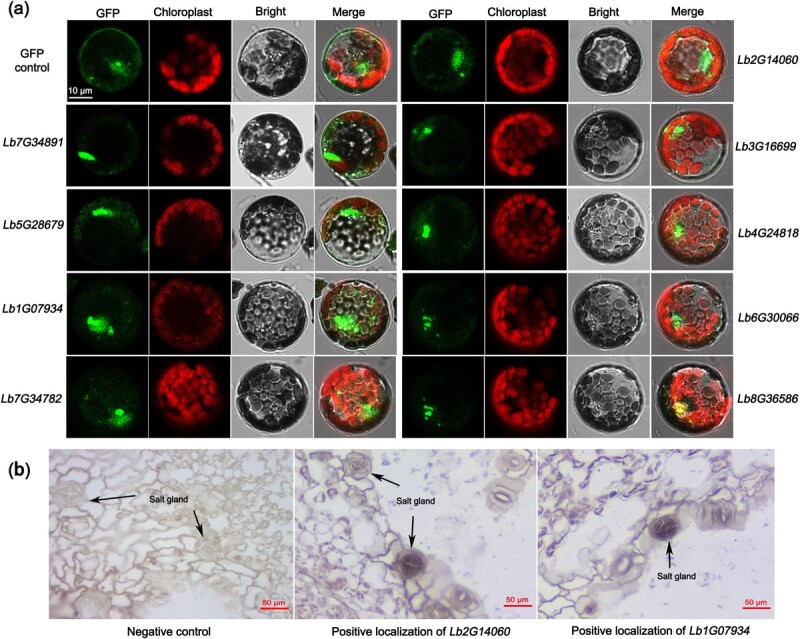
Localization of bHLH gene expression. **a** Positioning of 35S::LbbHLH-GFP in *Arabidopsis* protoplasts. The GFP-LbbHLH recombinant proteins were expressed only in the nucleus. Scale bar = 10 μm. **b** Negative control: the probe could not detect any transcripts and did not hybridize with any nucleic acid sequence. Localization of bHLH genes: LbbHLH transcripts were detected by an antisense probe (labeled with digoxin) that produces a blue–purple color. Scale bar = 50 μm.

We inserted the coding sequences of these nine genes into pCAMBIA1300 to generate GFP fusion proteins driven by the 35S promoter. We transferred these recombinant plasmids into *Arabidopsis* protoplasts by PEG-mediated transformation and examined fluorescent signals after 16 h of incubation. Several fusion proteins were expressed in the nucleus only, whereas signals from the GFP control were discovered in both the plasma membrane and the nucleus (Fig. 3a). The previously identified bHLH protein encoded by *Lb1G04899* was also found in the nucleus. These phenomena indicate that, to regulate the initial transcription and replication of *L. bicolor*, LbbHLHs exercise a function in the nucleus, even related to the early development of epidermal structures.

*In situ* hybridization was used to detect the localization of the above nine genes to test whether they were positioned in the salt glands. We detected distinct hybridization signals of *Lb1G07934* and *Lb2G14060* in salt glands, compared with the negative control. However, signals from the other seven genes were not detected in the salt glands (Fig. 3b). Furthermore, given that *Lb1G07934* showed high expression during salt gland development and NaCl treatment, *Lb1G07934* was selected for functional verification in salt gland development.

### Knockout of bHLH gene *Lb1G07934* enhances salt tolerance

**Figure 4:**
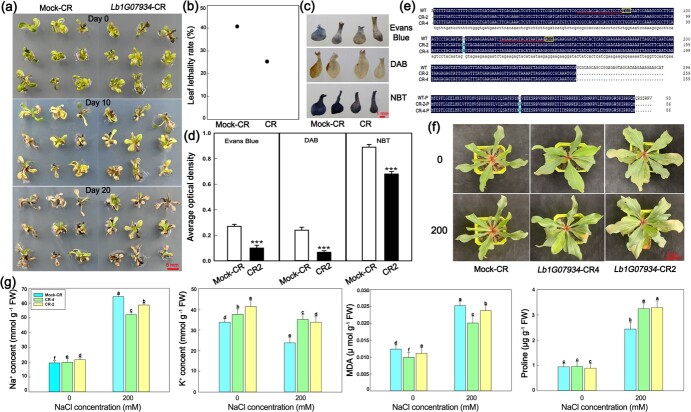
Growth status and indicators of CR lines of *L. bicolor*. **a** Growth status of transgenic plants growing in 200 mM NaCl medium at 0, 10, and 20 days. Scale bar = 5 mm. **b** Leaf lethality rates of transgenic lines. Data are means ± standard deviation of nine replicates. **c** Staining of leaves of regenerated lines. Scale bar = 1 mm. **d** Statistics of the degree of staining. Data are means ± standard deviation of nine replicates. SPSS was used to determine the statistical significance of the data in the *t*-test. ^***^P < 0.001. **e** Sequence alignment of *Lb1G07934* from the CRISPR mutants CR-2 and CR-4, showing DNA alignments. Red lines indicate the CRISPR target sites; yellow boxes indicate the PAM sequence. **f** Appearance comparison of three lines before and after treatment with 200 mM NaCl. Scale bar = 5 cm. **g** Na^+^, K^+^, MDA, and proline contents in three lines before and after 200 mM NaCl treatment. Data are means ± standard deviation of three replicates. SPSS was used to determine the statistical significance of the data. Different letters indicate significant differences (p = 0.05; Duncan’s multiple range test).

**Figure 5:**
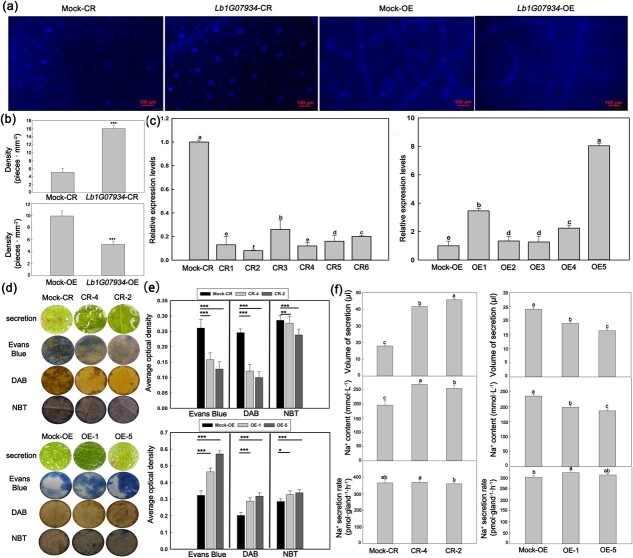
Appearance of salt glands and measurement of salt secretion in different *Lb1G07934* lines. **a** Phenotypes of salt glands in Mock-OE, Mock-CR, OE, and CR lines. **b** Density of salt glands in Mock-OE, Mock-CR, OE, and CR lines. Scale bar = 100 μm. Data are means ± standard deviation of 10 replicates. **c** Expression levels of *Lb1G07934* in *Lb1G07934*-CR and *Lb1G07934*-OE lines. The Mock-CR and Mock-OE lines were used as a control. **d** Secretion status and DAB, Evans Blue, and NBT staining of leaf discs after 24 h of treatment. **e** Quantitative analysis of DAB, Evans Blue, and NBT staining. Data are means ± standard deviation of three replicates. SPSS was used to determine the statistical significance of the data (**b** and **e**) in the *t*-test. ^*^P < 0.05; ^**^P < 0.01; ^***^P < 0.001. **f** Statistics of the volume of secretions, Na^+^ contents in secretions, and the rate of Na^+^ secretion by a single salt gland. SPSS was used to determine the statistical significance of the data (**c** and **f**) in Duncan’s multiple range test (*P* = 0.05).

In order to investigate the role of the key candidate gene in salt gland development and salt resistance, we first generated *Lb1G07934* knockout lines (*Lb1G07934*-CR lines; Fig. 4) via CRISPR-Cas9-mediated gene editing and constructed overexpression lines (*Lb1G07934*-OE lines; Fig. 5). We transferred *Lb1G07934*-CR lines to culture medium containing NaCl and compared their growth status before and after NaCl treatment. The *Lb1G07934*-CR lines showed stronger resistance to NaCl (Fig. 4a) and lower leaf mortality rates (Fig. 4b) than the Mock-CR lines.

DAB, Evans Blue, and NBT staining of the leaves of the two lines was performed (Fig. 4c). The Mock-CR lines showed a strong staining degree (indicating cell death), whereas the *Lb1G07934*-CR lines suffered less damage under salt stress (Fig. 4d). Therefore, the presence of numerous salt glands under salt stress promotes the efflux of excessive Na^+^ from the plant, thereby improving salt resistance.

After further identifying the mutation sites of CR2 and CR4 (Fig. 4e), grown-up plants were used for NaCl treatment to determine their salt tolerance. In general, *Lb1G07934*-CR lines behaved better than Mock-CR (Fig. 4f). In detail, the contents of Na^+^, MDA, and proline of the leaves all increased after NaCl treatment, while the content of K^+^ decreased (Fig. 4g). However, Na^+^ and MDA contents of CR lines were lower than Mock-CR after treatment, yet the contents of K^+^ and proline were opposite, which indicated that knockout of *Lb1G07934* can significantly increase salt tolerance.

### *Lb1G07934* affects salt resistance by influencing salt gland number and secretion ability of leaf

The *Lb1G07934*-CR lines had more salt glands than Mock-CR lines (Fig. 5a), while fewer salt glands existed in the overexpression lines than Mock-OE lines (Fig. 5a). The *Lb1G07934*-CR lines also had a greater salt gland density than *Lb1G07934*-OE lines (Fig. 5b), indicating that *Lb1G07934* can negatively regulate salt gland development.

Using the Mock-CR as a control, we examined the expression levels of *Lb1G07934* in six *Lb1G07934*-CR lines cultivated in nutrient soil (Fig. 5c). Lines CR-4 and CR-2 showed the lowest expression levels of this gene and were used to measure secretion ability. The secretory vesicles of CR-2 and CR-4 were significantly larger than those of Mock-CR (Fig. 5d). CR lines secreted almost twice as much liquid substance as Mock-CR, indicating that they have stronger secretion ability. The Na^+^ contents in the secretions were higher in CR-2 and CR-4, but the secretion ability of a single salt gland showed little difference among lines (Fig. 5f). When DAB, Evans Blue, and NBT staining of leaf discs incubated with NaCl was performed, CR-2 and CR-4 showed lighter staining than Mock-CR (Fig. 5e), indicating that they suffered less damage from salt stress. In short, the knocking out of *Lb1G07934* enhanced the efflux capacity of the plants by inducing the production of more salt glands, thereby promoting Na^+^ efflux and enhancing salt tolerance.

In order to verify the function of *Lb1G07934* again in salt gland development and salt resistance, the salt gland number and salt resistance of overexpression lines were analyzed to validate the results of CRISPR lines. With Mock-OE as a control, *Lb1G07934*-OE1 and *Lb1G07934*-OE5, with high expression levels of *Lb1G07934*, were chosen to measure salt secretion ability (Fig. 5c). Contrary to the CR lines, the OE lines had smaller secretory vesicles and less liquid substance than Mock-OE (Fig. 5d). Moreover, the Na^+^ content in the secretions of OE-1 and OE-5 was lower than that of Mock-OE, the secretion ability of individual salt glands did not differ significantly (Fig. 5f). When DAB, Evans Blue, and NBT staining of leaf discs incubated with NaCl was performed, OE-1 and OE-5 showed more obvious staining than Mock-OE (Fig. 5d and e), indicating that they suffered more severe damage from salt stress. In short, the overexpression of *Lb1G07934* reduced the efflux capacity of the plants by inducing the production of fewer salt glands, thereby reducing Na^+^ efflux and salt tolerance.

The knockout of *Lb1G0793*4 did not promote the formation of salt glands with multiple foci, but it increased the number of salt glands, indicating that *Lb1G07934* is not the decisive gene controlling salt gland development. The bHLH protein encoded by *Lb1G07934* likely interacts with other transcription factors (WD40, MYB, and so on) and forms complexes to function together. To explore these interactions, we constructed a diagram of a possible protein interaction network (Supplementary Data Fig. S2). This suggested that the protein encoded by Lb1G07934 might act in combination with other proteins, such as those encoded by Lb1G05095 and Lb1G05945, to regulate salt gland development and salt secretion. We plan to perform additional studies in the near future to better reveal the influences of bHLH genes in plant epidermal structure formation and stress mitigation.

## Discussion

The current study of the bHLH family and the validation of key genes indicated that some bHLH family members are closely related to the development of salt glands and salt tolerance. In particular, one bHLH gene, *Lb1G07934*, was proposed to participate in salt gland development and salt secretion by *in situ* hybridization, gene knockout and overexpression. Here, the reports of bHLH family and the functional identification of gene *Lb1G07934* made the groundwork for greater exploration of the functions and regulatory mechanisms of bHLH family genes, and they paved bridge for the discovery and application of salt tolerance genes in *L. bicolor*.

### Key genes for salt gland development may exist in the bHLH family

*Limonium bicolor* contains a large number of genes with high homology with *Arabidopsis*, such as *TRANSPARENT TESTA GLABRA1* (*TTG1*), *CAPRICE* (*CPC*), and *TRIPTYCHON* (*TRY*), with homology of up to 40%. Unfortunately, all bHLH genes in *L. bicolor* have low homology (<30%) with *AtGL3* [28], except for a key gene for salt gland development called *LbbHLH* (Lb1G04899, homology of 30–40%) [29]. LbbHLH can interact with LbTTG1 to inhibit the production of salt glands in the epidermal structure of *L. bicolor* [28], which was different from the role of AtGL3 in *Arabidopsis* in initiating trichome formation [30–32]. Thus, the roles of bHLH family genes need to be investigated in more detail, which is relevant to the study of salt glands.

Several candidate factors were selected with high homology with LbbHLH from the bHLH family in order to find more key genes related to salt gland development in *L. bicolor*. Interestingly and fortunately, another bHLH gene was verified in the current study to participate in salt gland development, and has functions similar to those of LbbHLH. This indicated that the essential genes involved in salt gland development may exist in the bHLH family, and also suggested that multiple genes take part in salt gland development.

### bHLH genes of *L. bicolor* are related to the development of epidermal structures, especially salt gland formation

The bHLH gene family is widely distributed in plants, with rich biological functions, such as promoting epidermal structure development in *Arabidopsis* [33, 34] and participating in plant responses to NaCl [35], among others. Thus, it is beneficial to explore the potential functions of bHLH genes in salt mitigation in *L. bicolor*. It is particularly necessary to clarify their upstream and downstream regulatory genes and to establish a complete functional relationship network for the use of salt tolerance genes in other plants, especially crops.

TDR INTERACTING PROTEIN 2 (TIP2), a bHLH-type transcription factor in rice, functions in the differentiation of the middle layer and tapetum of the anther by regulating the expression of *TAPETUM DEGENERATION RETARDATION* (*TDR*) and *ETERNAL TAPETUM 1* (*EAT1*) [36]. Watermelon (*Citrullus lanatus*) *Abnormal Tapetum 1* (*ClATM1*), a sterility gene of the male in this species, encodes a bHLH protein that acts to regulate anther formation and differentiation [37]. In sweet sorghum (*Sorghum bicolor*), SbbHLH85 plays a key role in root by increasing the length and number of root hairs through the abscisic acid and auxin signaling pathways [38]. Abundant *GhDEL65* transcripts, encoding a bHLH protein in cotton (*Gossypium hirsutum*), were detected in ovules after anthesis and were abundant in fibers, suggesting that GhDEL65 functions in early fiber development in cotton [39]. ZmbHLH genes are expressed highly in maize during root, leaf, and seed development and might participate in multiple plant growth and developmental processes [40]. In physic nut (*Jatropha curcas*), bHLH genes other than *JcbHLH84* are expressed in various parts, implying they play crucial roles in the overall growth of plants [41]. Consequently, bHLH proteins function in epidermal structure development in many species.

In *L. bicolor*, bHLH genes are likewise closely related to plant development, especially the formation and differentiation of epidermal structures such as salt glands. *Lb1G04899* is positioned in an important node of the protein interaction network diagram (Supplementary Data Fig. S2), providing further evidence for our prior suspicion that this gene participates in salt gland development [28, 29]. In addition, we showed here that the bHLH gene *Lb1G07934* participates in salt gland development by *in situ* hybridization and gene knockout. Together, these results suggest that multiple bHLH transcription factors in *L. bicolor* are related to the generation of plant epidermal structures, especially salt glands.

### The *L. bicolor* bHLH transcription factor family functions in salt tolerance

In orchard grass (*Dactylis glomerata*), bHLH participates in abiotic stress responses by binding to MYC elements; for example, DgbHLH46 and DgbHLH128 enhance tolerance of salt stress [35]. A kind of non-DNA binding protein, paclobutrazol resistances (PREs), participate in salt responses in *Arabidopsis*, and their expression significantly increased under salt treatment. 35S:*PRE6* transgenic sorghum plants show increased salt tolerance, and PREs might play redundant roles in regulating salt responses [42]. In physic nut, eight bHLH genes are closely related to salinity and others respond to drought stress [41]. In *Hibiscus hamabo* Sieb. et Zucc., most HhbHLH genes contain *cis* elements closed to development, differentiation, and abiotic stress responses [43]. Exogenously expressing *HhbHLH2* increased the adaptation of *Arabidopsis* to salt and drought stress. Most PbrbHLHs in Chinese white pear (*Pyrus bretschneideri*) are closely related to responses to drought and cold stress [14]. In ‘Golden Delicious’ apple (*Malus × domestica* Borkh.), MdbHLH proteins might function in resistance to water scarcity stress and salt stress [44]. Overexpressing *ZjICE2* conferred abiotic stress resistance in transgenic *Arabidopsis* [45]. Therefore, bHLH genes in *L. bicolor* likely respond to abiotic stress, especially salt stress. The enhanced growth status of *Lb1G07934*-CR lines under 200 mM NaCl treatment confirms this notion. The degree of tissue staining confirms that the *Lb1G07934*-CR lines experienced less stress damage than the control. The physiological indicators of the grown-up knockout lines after NaCl treatment also show increased salt tolerance due to the enhanced salt gland development after *Lb1G07934* knockout.

In our study, we identified 187 bHLH genes in the *L. bicolor* genome and performed bioinformatics analysis. We examined their expression patterns based on previous transcriptome data. We confirmed the possible role of the bHLH gene *Lb1G07934* in salt glands in *L. bicolor* through salt tolerance tests of CR-*Lb1G07934* lines. Our results lay the foundation for further studies of the influences of bHLH genes in salt tolerance in the halophyte *L. bicolor*.

At present, there is no report on the specific bHLH gene known to regulate salt gland development in other halophyte species, making it difficult to find homologous and unique genes for salt gland development. Most studies about *L. bicolor* remain at the developmental level of the epidermal structure of heterologous overexpression plants [20, 46–48], and there has been little research into the roles of *L. bicolor* genes in salt gland generation and salt discharge. Earlier, we confirmed by *in situ* hybridization that the *L. bicolor* gene *Lb1G04899* (*LbbHLH*) is expressed in salt glands [29]; what is more, *Lb1G04899* participates in salt gland differentiation and responds to salt stress [28], laying a foundation for further exploration of the effects of bHLH genes in *L. bicolor* salt tolerance. Further research in this area is greatly needed, and in particular optimizing the genetic transformation system in this halophyte species will be a crucial next step.

## Conclusion

187 bHLH family proteins were identified in L.bicolor, which distributed on 8 different chromosomes and are closer to Fagopyrum tataricum. These genes are expressed in various stages of L.bicolor and early stages of NaCl treatment, probably related to the development and responses to salt stress. Nine bHLH genes play a role in the nucleus, we believe that they have great potential to regulate the development of L.bicolor and participate in salt resistance. In situ hybridization showed that Lb1G07934 was expressed in the salt gland, which further explained that the bHLH family genes of L.bicolor might participate in the leaves development regulation and salt secretion. The salt tolerance of bHLH transgenic lines also verified this function. This study provides a basis for further exploring the function and regulation mechanism of bHLH family genes, also lays a foundation for the discovery and application of salt tolerant genes in L.bicolor.

## Materials and methods

### Plant seeds and cultivation conditions

Wild-type *L. bicolor* seeds were harvested from Dongying saline land (37°30′ N, 117°86 E), Shandong Province, China. The seeds were thoroughly dried and stored in a regular low-temperature refrigerator for further use. To obtain aseptic seedlings, the dried seeds were cleaned with 75% ethanol on a 180-rpm shaking table for 5 min and further disinfected in NaClO (6%, v/v) for 19 min. With sterile distilled water, the seeds were cleaned four or five times, soaked for 20 min until the seed coats began to fall off, and placed on MS (Murashige and Skoog) medium solidified with 1% agar powder in a sterile culture dish. Plant seedlings were cultured at a light intensity of 580 μmol/m^2^/s (17-h photoperiod), a humidity of 68%, and 29°C/22°C (day/night) temperatures.

*Arabidopsis* Columbia-0 seeds were cleaned twice with 75% ethanol (10 min in total) and twice with 95% ethanol (4 min each time). The surface disinfection process was performed using an oscillator. The seeds of *Arabidopsis* were placed on half-strength MS medium and vernalized in a regular low-temperature refrigerator (4°C) for 2–3 days prior to culture. The seeds were germinated at 23°C/19°C (day/night) under a 17-h/7-h light/dark cycle with a luminosity of 155 μmol/m^2^/s and 68% humidity. Seedlings germinated in the medium for 7 days, then were transferred to pots containing nutrient soil.

### Identification of bHLH genes in *L. bicolor*

On the basis of the genome annotation and sequence of *L. bicolor* (BioProject number PRJNA753199), all bHLH genes were identified by deep mining based on the appearance of the bHLH region. Using the Pfam_scan program, the protein sequences containing the conserved bHLH domain (PF00010.25 bHLH and PF14215.5 bHLH) were screened as the result of preliminary screening in the Pfam database. Furthermore, in-depth confirmation was conducted using SMART. No potential duplicated genes emerged from this screening. In order to confirm the authenticity of the screened bHLHs, all sequences were compared in the NR, TrEMBL, Swiss-Prot, KEGG, and KOG databases using the diamond BLAST (threshold set to evaluate <1e−5) program. Finally, manual validation of the bHLH genes was confirmed through the NCBI BLAST program.

### Bioinformatic analysis

By using ExPASy, which contains ProtParam (https://web.expasy.org/protparam/), the MW and pI of the bHLH proteins were predicted. Previous studies have identified genes in *L. bicolor* that regulate the initiation of *Arabidopsis* trichomes, and speculated that genes that controlled salt gland differentiation evolved in *Arabidopsis* trichome formation [23]. To analyze their evolutionary relationships, the full-length bHLH proteins of *L. bicolor* and *Arabidopsis* were compared by the MUSCLE program in MEGAX. A maximum-likelihood tree was formed according to the alignment results. Finally, the maximum-likelihood tree was optimized by using the online tool iTOL (https://itol.embl.de/). Information about exons and introns in bHLH family members was extracted from the gff file. Information about motifs was extracted from the pfam annotation results. Using the Gene Structure Display Server (GSDS) program, the composition of genes and motif distribution map were drawn. The conserved amino acid motifs of the bHLH proteins were analyzed with the MEME suite (http://meme-suite.org/tools/meme).

The promoter sequences were submitted to the PlantCARE database to explore the *cis* elements in the bHLH gene promoter regions. TBtools and One Step MCScanX were used for the synteny analysis of *L. bicolor* and the bHLH genes of *Arabidopsis*, and the results were visualized using Circos. Meanwhile, interspecific analysis was performed after extracting collinear blocks between *L. bicolor* and *Arabidopsis*, *Beta vulgaris*, and *Fagopyrum tataricum* via JCVI. bHLH family members were selected in these collinear blocks and visualized using the JCVI drawing subroutine. The STRING protein interaction database was used to calculate the genome-wide protein interaction matrix, and non-bHLH proteins that interact with bHLH genes were extracted to form the bHLH protein interaction matrix.

### Construction of expression vectors

A sufficient number of first true leaves distributed among the A–E stages (different developmental stages) were collected from *L. bicolor* and ground to a power in a mortar filled with liquid nitrogen for total RNA extraction. Total RNA of the leaves was extracted through a FastPure Plant Total RNA Isolation kit (RC411-C1; Vazyme Biotech Co., Ltd, Nanjing, China). In accordance with the manufacturer’s instructions, after reverse transcription, RNA generated cDNA with a SPARKscript II RT Plus Kit (With gDNAEraser) (Shandong Sparkjade Biotechnology Co., Ltd). Using the cDNA as a template, two complementary strands of nine bHLH genes were synthesized using specific primers (e.g. *Lb7G34891*-S and *Lb7G34891*-A) (Supplementary Data Table S3) designed by Primer 5.0 to complete the amplification of the full gene sequence.

Using primers (e.g. *Lb7G34891*-OE-S and *Lb7G34891*-OE-A) containing the pCAMBIA1300 homologous arm, the bHLH gene fragments carrying SalI digestion sites were amplified. At the same time, the pCAMBIA1300 plasmid was digested and become to a linear vector through digestion with SalI to construct the pCAMBIA1300-*Lb1G07934* vector. According to the manufacturer’s instructions, each bHLH gene segment with a SalI site was connected to the non-circular pCAMBIA1300 vector using a ClonExpress II One Step Cloning Kit (Vazyme Biotech Co., Ltd, China), and homologous recombination was performed to generate recombinant GFP proteins, which were used to analyze the subcellular localizations of the bHLHs.

### Analysis of subcellular localization

The analysis of subcellular localization involved protoplast isolation, transformation with plasmid DNA, and protoplast culture. To generate *Arabidopsis* protoplasts, fully stretched *Arabidopsis* leaves were treated with Cellulose R10, Macerozyme R10, and pectinase. A large number of protoplasts were released, which were filtered, washed, and centrifuged subsequently. A construct encoding recombinant GFP protein was transformed into the protoplasts, while the negative control (GFP alone) was also transformed [28, 49, 50]. After 12–16 h of culture at 22°C, the expression of the fusion protein reached its highest level. Using a TCS S8MP two-photon laser-scanning confocal microscope (Leica, Germany), the GFP fluorescent signal was detected.

### Analysis of *in situ* hybridization

To explore the regions of bHLH gene expression in *L. bicolor*, developing true leaves were isolated from *L. bicolor* and dehydrated through ethanol after being soaked in paraformaldehyde (4%) and wrapped in paraffin, and finally subjected to *in situ* hybridization. Tissue sections (9 μm) were treated with proteinase K and hybridized in a solution with 6 ng/μl for 12–16 h at 36°C. The *Lb2G14060* probe (5′-DIG-GGGUAAACGGGCGAAGCACUUGGUUGAUC-3′, purified by HPLC) which, labeled with digoxin and *Lb1G07934* probe (5′-DIG-AGAGGCGAAGUUAUCAUCAAUGACGACAGA-3′, purified by HPLC), appeared blue–purple.

### Expression patterns during leaf development and NaCl treatment

The expression characteristics of the identified bHLH family members were analyzed based on our previous transcriptome data. Transcriptome data (BioProject number PRJNA752802) composed of leaves of *L. bicolor* at different development stages (A–E) were used to quantify the expression status of all bHLH family genes. To verify the salt responses of all bHLH genes, transcriptome data from *L. bicolor* leaves at different time points of NaCl treatment (0, 12, 24, 48, and 72 h) were also analyzed (PRJNA752940).

The data from RNA-seq were subjected to quality filtering (completed by the fastq program with default parameters) and compared with the genome using the HISAT2 program. FPKM of each gene was calculated by calling the Cuffquant and Cuffnorm program in Cufflinks. The FPKM values of bHLH genes were extracted and drawn in a heat map after normalizing the FPKM by row with the *z*-score.

### Construction of CRISPR-cas9 vector

The pHEC401 vector was digested with BsaI to obtain the linearized product. PCR amplification was carried out using pCBC-DT1T2 as a template with RNA primers (*Lb1G07934*-primer1, *Lb1G07934*-primer2, *Lb1G07934*-primer3, and *Lb1G07934*-primer4) for *Lb1G07934*. The PCR products were ligated to the pHEC401 vector using T4 DNA ligase to generate the corresponding CRISPR knockout construct, which was used to produce knockout plants [28, 51].

### Generation of overexpression lines and CRISPR lines of *Lb1G07934*

The knockout vector pHEC401-*Lb1G07934* and the recombinant pCAMBIA1300-*Lb1G07934* vector were transformed into *L. bicolor* to generate knockout plants (CR) and overexpressing lines (OE) according to a published transformation protocol for *L. bicolor* [25]. In brief, the vectors were introduced into *L. bicolor* cotyledons via *Agrobacterium tumefaciens* (strain EHA105)-mediated transformation. The cotyledons were transferred to budding medium containing 6-benzylaminopurine (6-BA) for redifferentiation and growth. Regenerated buds were separated from explants and placed in rooting medium including indole-3-butyric acid (IBA) and 8 mg/l hygromycin for selection and rooting. A pHEC401 vector carrying *Cas9* without RNA primers was transformed into plants as a negative control to exclude the possible influence of gene editing itself, and the resulting plants were named Mock-CR. pCAMBIA1300 empty vector was transformed into plants as a positive control, and the resulting plants were named Mock-OE.

### Observation of salt glands and determination of salt tolerance of transgenic plants

The first fully extended true leaves of transgenic plants were removed from rooting medium and placed in fixation solution (3/4 ethanol and 1/4 acetic acid). Following overnight incubation, the leaves were placed in decolorization solution (70% ethanol, v/v) to completely remove the chlorophyll and observed under a fluorescence microscope (Eclipse 80i, Nikon, Japan). Salt glands were observed under 325–385 nm UV excitation. Salt gland density (mm^2^) was calculated as total number of salt glands in a leaf/total area of a leaf × 100%. Two lines were examined, with 10 leaves examined per line.

Transgenic plants were transplanted into 200 mM NaCl-containing MS medium; Mock-CR seedlings were used as controls. We conducted nine biological replicates for each line for the salt tolerance experiment using whole plants. The growth status was recorded by taking photos before and after NaCl treatment. The leaf lethality rate was calculated in each line with nine replications.

Leaves of two lines growing on 200 mM NaCl medium were stained by soaking in 1 mg/ml diaminobenzidine (DAB), nitro blue tetrazolium (NBT) at 0.5 mg/ml, and 0.25% (w/v) Evans Blue for 10, 4, and 1 h, respectively. Before observation, the leaves were decolorized in 75% ethanol to completely remove the chlorophyll. Subsequently, the staining pattern was photographed, and quantitative analysis of the degree of staining was performed using ImageJ.

### Measurement of salt secretion in knockout and overexpression lines

The seedlings selected from screening medium were transferred to nutrient soil and grown for 1 month. Total RNA of six knockout lines and five overexpression lines was extracted and transformation efficiency was analyzed by measuring *Lb1G07934* expression by RT–qPCR (with Mock-CR used as a control) with a 2× T5 Fast qPCR Mix (SYBR Green I) (Tsingke Biological Technology, Beijing, China).

The lines with the lowest *Lb1G07934* expression levels (CR-4 and CR-2) and highest *Lb1G07934* expression levels (OE-1 and OE-5) were selected for salt secretion assays using leaf discs. Leaf discs (covered in mineral oil) with a diameter of 1 cm were placed horizontally on a culture dish containing 200 mmol of sodium chloride and incubated overnight. The secretions were collected and the volume of the secretions was measured. After dilution of the secretion 200-fold, the Na^+^ content was measured and one salt gland’s secretion rate was calculated. A single salt gland’s Na^+^ discharge rate was calculated as (Na^+^ content in secretion × volume of secretion/total number of salt glands in one leaf disc)/24. Subsequently, the treated leaf discs were stained with DAB, Evans Blue, and NBT, and the degree of staining was quantified. Ten leaf discs (biological repeats) were examined for each line.

### Identification of knockout sites

After extracting mRNA of CR-2, CR-4, and wild-type, the cDNAs were used as templates for PCR cloning under the guidance of primers JD-S (5-ATGTTTGATCCTTTTCTGGG-3) and JD-A (5-CATGCTTCTTTCTTATGCTACT-3), and then the PCR products were sequenced to identify the knockout sites.

### Determination of physiological indicators of grown-up CRISPR plants

The mature CRISPR plants (Mock-CR, CR-2 and CR-4) were transplanted into soil and cultured for 3 months before treatment with 200 mM NaCl to further determine their salt resistance. After treatment for 1 week, the leaves of all lines were collected for the determination of Na^+^, K^+^, malondialdehyde (MDA), and proline as reported [52, 53]. Three replicates were repeated in each indicator.

### Statistical analysis

The SPSS program was used to determine the statistical significance of the data in Duncan’s multiple range test (*P* = 0.05) and the *t*-test. Mean comparison procedures and ANOVA with orthogonal contrasts were used to check significant differences between groups.

## Supplementary Material

Web_Material_uhae036

## Data Availability

The genome and transcriptome data underlying this article are available in NCBI at https://www.ncbi.nlm.nih.gov/bioproject/, and can be accessed with BioProject numbers PRJNA753199, PRJNA752802, and PRJNA752940. The data underlying this article will be shared on reasonable request to the corresponding author.

## References

[1] Yuan J , Zhang S, Liu Z. et al. Cloning and phylogenetic analysis of an amphioxus myogenic bHLH gene AmphiMDF. Biochem Biophys Res Commun. 2003;301:960–7 12589806 10.1016/s0006-291x(03)00081-0

[2] Wang L , Xiang L, Hong J. et al. Genome-wide analysis of bHLH transcription factor family reveals their involvement in biotic and abiotic stress responses in wheat (*Triticum aestivum* L.). 3 Biotech. 2019;9:236 10.1007/s13205-019-1742-4 PMC6536565 31139551

[3] Sun W , Xiu J, Ma Z. et al. Basic helix–loop–helix (bHLH) gene family in Tartary buckwheat (*Fagopyrum tataricum*): genome-wide identification, phylogeny, evolutionary expansion and expression analyses. *Int J Biol Macromol*. 2020;155:1478–90 31734362 10.1016/j.ijbiomac.2019.11.126

[4] Bailey PC , Martin C, Toledo-Ortiz G. et al. Update on the basic helix-loop-helix transcription factor gene family in *Arabidopsis thaliana*. *Plant Cell*. 2003;15:2497–502 14600211 10.1105/tpc.151140 PMC540267

[5] Zhang Z , Chen J, Liang C. et al. Genome-wide identification and characterization of the bHLH transcription factor family in pepper (*Capsicum annuum* L.). *Front Genet*. 2020;11:570156 33101390 10.3389/fgene.2020.570156 PMC7545091

[6] Wang R , Zhao P, Kong N. et al. Genome-wide identification and characterization of the potato bHLH transcription factor family. *Genes (Basel)*. 2018;9:54 29361801 10.3390/genes9010054 PMC5793205

[7] Li J , Wang T, Han J. et al. Genome-wide identification and characterization of cucumber bHLH family genes and the functional characterization of CsbHLH041 in NaCl and ABA tolerance in *Arabidopsis* and cucumber. *BMC Plant Biol*. 2020;20:272 32527214 10.1186/s12870-020-02440-1 PMC7291561

[8] Kavas M , Baloglu MC, Atabay ES. et al. Genome-wide characterization and expression analysis of common bean bHLH transcription factors in response to excess salt concentration. *Mol Gen Genomics*. 2016;291:129–43 10.1007/s00438-015-1095-6 26193947

[9] Shen W , Cui X, Li H. et al. Genome-wide identification and analyses of bHLH family genes in *Brassica napus*. *Can J Plant Sci*. 2019;99:589–98

[10] Groszmann M , Bylstra Y, Lampugnani ER. et al. Regulation of tissue-specific expression of SPATULA, a bHLH gene involved in carpel development, seedling germination, and lateral organ growth in *Arabidopsis*. *J Exp Bot*. 2010;61:1495–508 20176890 10.1093/jxb/erq015 PMC2837263

[11] Ludwig SR , Habera LF, Dellaporta SL. et al. Lc, a member of the maize R gene family responsible for tissue-specific anthocyanin production, encodes a protein similar to transcriptional activators and contains the myc-homology region. *Proc Natl Acad Sci USA*. 1989;86:7092–6 2674946 10.1073/pnas.86.18.7092 PMC298000

[12] Gao C , Sun J, Wang C. et al. Genome-wide analysis of basic/helix-loop-helix gene family in peanut and assessment of its roles in pod development. *PLoS One*. 2017;12:e0181843 28750081 10.1371/journal.pone.0181843 PMC5531549

[13] Zhu L , Zhao M, Chen M. et al. The bHLH gene family and its response to saline stress in Jilin ginseng, *Panax ginseng* C.A. Meyer. *Mol Gen Genomics*. 2020;295:877–90 10.1007/s00438-020-01658-w 32239329

[14] Dong H , Chen Q, Dai Y. et al. Genome-wide identification of PbrbHLH family genes, and expression analysis in response to drought and cold stresses in pear (*Pyrus bretschneideri*). *BMC Plant Biol*. 2021;21:86 33563216 10.1186/s12870-021-02862-5 PMC7874673

[15] Kou X , Xiong C, Wang D. et al. Comparative analysis of bHLH transcription factors in five Rosaceae species, and expression analysis of PbbHLHs in response to drought stress in pear. *Res Sq*. 2020;

[16] Wei K , Chen H. Comparative functional genomics analysis of bHLH gene family in rice, maize and wheat. *BMC Plant Biol*. 2018;18:309 30497403 10.1186/s12870-018-1529-5 PMC6267037

[17] Munns R , Tester M. Mechanisms of salinity tolerance. *Annu Rev Plant Biol*. 2008;59:651–81 18444910 10.1146/annurev.arplant.59.032607.092911

[18] Singh A . Soil salinization management for sustainable development: a review. *J Environ Manag*. 2021;277:111383 10.1016/j.jenvman.2020.111383 33035935

[19] Litalien A , Zeeb B. Curing the earth: a review of anthropogenic soil salinization and plant-based strategies for sustainable mitigation. *Sci Total Environ*. 2020;698:134235 31783465 10.1016/j.scitotenv.2019.134235

[20] Fang T , Rao Y, Wang M. et al. Characterization of the SWEET gene family in longan (*Dimocarpus longan*) and the role of DlSWEET1 in cold tolerance. *Int J Mol Sci*. 2022;23:8914 36012186 10.3390/ijms23168914 PMC9408694

[21] Flowers TJ , Colmer TD. Salinity tolerance in halophytes. *New Phytol*. 2008;179:945–63 18565144 10.1111/j.1469-8137.2008.02531.x

[22] Yuan F , Leng B, Wang B. Progress in studying salt secretion from the salt glands in recretohalophytes: how do plants secrete salt? *Front Plant Sci*. 2016;7:977 27446195 10.3389/fpls.2016.00977 PMC4927796

[23] Yuan F , Lyu MJA, Leng BY. et al. Comparative transcriptome analysis of developmental stages of the *Limonium bicolor* leaf generates insights into salt gland differentiation. *Plant Cell Environ*. 2015;38:1637–57 25651944 10.1111/pce.12514

[24] Yuan F , Lyu MJA, Leng BY. et al. The transcriptome of NaCl-treated *Limonium bicolor* leaves reveals the genes controlling salt secretion of salt gland. *Plant Mol Biol*. 2016;91:241–56 26936070 10.1007/s11103-016-0460-0

[25] Yuan F , Chen M, Yang J. et al. A system for the transformation and regeneration of the recretohalophyte *Limonium bicolor*. *In Vitro Cell Dev Biol Plant*. 2014;50:610–7

[26] Zou H , Leng B, Gao Y. et al. The MYB transcription factor LbCPC of *Limonium bicolor* negatively regulates salt gland development and salt tolerance. *Environ Exp Bot*. 2023;209:105310

[27] Zhou Y , Zhang H, Ren Y. et al. The transmembrane protein LbRSG from the recretohalophyte *Limonium bicolor* enhances salt gland development and salt tolerance. *Plant J*. 2024;117:498–515 37856574 10.1111/tpj.16505

[28] Yuan F , Wang X, Zhao B. et al. The genome of the recretohalophyte *Limonium bicolor* provides insights into salt gland development and salinity adaptation during terrestrial evolution. *Mol Plant*. 2022;15:1024–44 35514085 10.1016/j.molp.2022.04.011

[29] Wang X , Zhou Y, Xu Y. et al. A novel gene LbHLH from the halophyte *Limonium bicolor* enhances salt tolerance via reducing root hair development and enhancing osmotic resistance. *BMC Plant Biol*. 2021;21:284 34157974 10.1186/s12870-021-03094-3 PMC8218485

[30] Gao C , Guo Y, Wang JLD. et al. *Brassica napus* GLABRA3-1 promotes anthocyanin biosynthesis and trichome formation in true leaves when expressed in *Arabidopsis thaliana*. *Plant Biol (Stuttg)*. 2018;20:3–9 10.1111/plb.12633 28940939

[31] Zhang Y , Zhu H, Shao C. et al. PaMYB82 from *Platanus acerifolia* regulates trichome development in transgenic *Arabidopsis*. *Plant Sci*. 2019;287:110177 31481209 10.1016/j.plantsci.2019.110177

[32] Gonzalez A , Zhao M, Leavitt JM. et al. Regulation of the anthocyanin biosynthetic pathway by the TTG1/bHLH/Myb transcriptional complex in *Arabidopsis* seedlings. *Plant J*. 2008;53:814–27 18036197 10.1111/j.1365-313X.2007.03373.x

[33] Zhao M , Morohashi K, Hatlestad G. et al. The TTG1-bHLH-MYB complex controls trichome cell fate and patterning through direct targeting of regulatory loci. *Development*. 2008;135:1991–9 18434419 10.1242/dev.016873

[34] Zhao M . Regulation of arabidopsis trichome patterning and anthocyanin biosynthesis by the TTG1-bHLH-MYB complex. PhD Thesis,. University of Texas at Austin; 2007:

[35] Lu X , Zhang H, Hu J. et al. Genome-wide identification and characterization of bHLH family genes from orchardgrass and the functional characterization of DgbHLH46 and DgbHLH128 in drought and salt tolerance. *Funct Integr Genomics*. 2022;22:1331–44 35941266 10.1007/s10142-022-00890-4

[36] Fu Z , Yu J, Cheng X. et al. The rice basic helix-loop-helix transcription factor TDR INTERACTING PROTEIN2 is a central switch in early anther development. *Plant Cell*. 2014;26:1512–24 24755456 10.1105/tpc.114.123745 PMC4036568

[37] Zhang R , Chang J, Li J. et al. Disruption of the bHLH transcription factor Abnormal Tapetum 1 causes male sterility in watermelon. Hortic Res. 2021;8:258 34848708 10.1038/s41438-021-00695-9 PMC8632879

[38] Song Y , Li S, Sui Y. et al. SbbHLH85, a bHLH member, modulates resilience to salt stress by regulating root hair growth in sweet sorghum. *Theor Appl Genet*. 2021;135:201–16 34633473 10.1007/s00122-021-03960-6

[39] Shangguan XX , Yang CQ, Zhang XF. et al. Functional characterization of a basic helix-loop-helix (bHLH) transcription factor GhDEL65 from cotton (*Gossypium hirsutum*). *Physiol Plant*. 2016;158:200–12 27080593 10.1111/ppl.12450

[40] Zhang T , Lv W, Zhang H. et al. Genome-wide analysis of the basic helix-loop-helix (bHLH) transcription factor family in maize. *BMC Plant Biol*. 2018;18:235 30326829 10.1186/s12870-018-1441-z PMC6192367

[41] Zhang L , Chen W, Liu R. et al. Genome-wide characterization and expression analysis of bHLH gene family in physic nut (*Jatropha curcas* L.). *PeerJ*. 2022;10:e13786 35966923 10.7717/peerj.13786 PMC9373979

[42] Zheng K , Wang Y, Wang S. The non-DNA binding bHLH transcription factor Paclobutrazol Resistances are involved in the regulation of ABA and salt responses in *Arabidopsis*. *Plant Physiol Biochem*. 2019;139:239–45 30921735 10.1016/j.plaphy.2019.03.026

[43] Ni L , Wang Z, Fu Z. et al. Genome-wide ana1ysis of basic he1ix-1oop-he1ix fami1y genes and expression ana1ysis in response to drought and sa1t stresses in *Hibiscus hamabo* Sieb. et Zucc. *Int J Mol Sci*. 2021;22:8748 34445454 10.3390/ijms22168748 PMC8395896

[44] Mao K , Dong Q, Li C. et al. Genome wide identification and characterization of apple bHLH transcription factors and expression analysis in response to drought and salt stress. *Front*. *Plant Sci*. 2017;8:480 10.3389/fpls.2017.00480 PMC5387082 28443104

[45] Zuo ZF , Kang HG, Hong QC. et al. A novel basic helix-loop-helix transcription factor, ZjICE2 from *Zoysia japonica* confers abiotic stress tolerance to transgenic plants via activating the DREB/CBF regulon and enhancing ROS scavenging. *Plant Mol Biol*. 2020;102:447–62 31898148 10.1007/s11103-019-00957-0

[46] Leng B , Wang X, Yuan F. et al. Heterologous expression of the *Limonium bicolor* MYB transcription factor LbTRY in *Arabidopsis thaliana* increases salt sensitivity by modifying root hair development and osmotic homeostasis. *Plant Sci*. 2021;302:110704 33288017 10.1016/j.plantsci.2020.110704

[47] Xu Y , Jiao X, Wang X. et al. Importin-β from the recretohalophyte *Limonium bicolor* enhances salt tolerance in *Arabidopsis thaliana* by reducing root hair development and abscisic acid sensitivity. *Front*. *Plant Sci*. 2021;11:582459 10.3389/fpls.2020.582459 PMC7838111 33519843

[48] Yuan F , Leng B, Zhang H. et al. A WD40-repeat protein from the recretohalophyte *Limonium bicolor* enhances trichome formation and salt tolerance in *Arabidopsis*. *Front*. *Plant Sci*. 2019;10:1456 10.3389/fpls.2019.01456 PMC6861380 31781150

[49] Yu G , Cheng Q, Xie Z. et al. An efficient protocol for perennial ryegrass mesophyll protoplast isolation and transformation, and its application on interaction study between LpNOL and LpNYC1. *Plant Methods*. 2017;13:46 28592987 10.1186/s13007-017-0196-0 PMC5460552

[50] Yoo S-D , Cho Y-H, Sheen J. *Arabidopsis* mesophyll protoplasts: a versatile cell system for transient gene expression analysis. *Nat Protoc*. 2007;2:1565–72 17585298 10.1038/nprot.2007.199

[51] Ma X , Zhang Q, Zhu Q. et al. A robust CRISPR/Cas9 system for convenient, high-efficiency multiplex genome editing in monocot and dicot plants. *Mol Plant*. 2015;8:1274–84 25917172 10.1016/j.molp.2015.04.007

[52] Ding M , Hou P, Shen X. et al. Salt-induced expression of genes related to Na^+^/K^+^ and ROS homeostasis in leaves of salt-resistant and salt-sensitive poplar species. *Plant Mol Biol*. 2010;73:251–69 20157764 10.1007/s11103-010-9612-9

[53] Patanè C , Cosentino SL, Romano D. et al. Relative water content, proline, and antioxidant enzymes in leaves of long shelf-life tomatoes under drought stress and rewatering. *Plants (Basel)*. 2022;11:3045 36432775 10.3390/plants11223045 PMC9699019

