# Supplementary material for: Genome-wide identification of bHLH transcription factors and functional analysis in salt gland development of the recretohalophyte sea lavender (Limonium bicolor)
Source: Hortic Res. 2024 Feb 2;11(4):uhae036. doi: 10.1093/hr/uhae036 (PMC11001596; doi:10.1093/hr/uhae036)
Supplement: Web_Material_uhae036 [file web_material_uhae036.zip › Table S3.docx]

Table S1 The primers used in this paper

| **Name** | **Oligonucleotide sequence** | **Role** |
| --- | --- | --- |
| *Lb7G34891-S* | 5- ATGATGGATGCTCACTTGCCACA -3 |  |
| *Lb7G34891*-A | 5- CTAGCAGTTGAGCGGATACCTCT -3 |  |
| *Lb5G28679*-S | 5-ATGGAATCATCGAGTTTCCCTC-3 |  |
| *Lb5G28679*-A | 5-TTAGTATGCCCCCGCGATCGGTA-3 |  |
| *Lb1G02274*-S | 5-ATGGTCGTGGACGCTTACAGC-3 |  |
| *Lb1G02274*-A | 5-CTATTCCGTAAACCCCGCTAAC-3 |  |
| *Lb1G07934*-S | 5-ATGTTTGATCCTTTTCTGGGTGA-3 |  |
| *Lb1G07934*-A | 5-CATCGGCGGTACACCAACC-3 |  |
| *Lb7G34782*-S | 5-ATGGTGATTCTTATTACTCTGA-3 |  |
| *Lb7G34782*-A | 5-CTACAAGAAGTGCAGCTCTGTC-3 | Full length amplification |
| *Lb2G14060*-S | 5-ATGGCTAATTTCAATACGAACT-3 |  |
| *Lb2G14060*-A | 5-TTACCTGCATCTCCCGCATGTGC-3 |  |
| *Lb3G16699*-S | 5-ATGTCTACGGACCAGCTTACTC-3 |  |
| *Lb3G16699*-A | 5-TCACATCTCCATCTTTAGACAA-3 |  |
| *Lb4G24818*-S | 5-ATGGCTGTTCCGGCTGCCGGAA-3 |  |
| *Lb4G24818*-A | 5-CTACAAGGAGTTGTTGGTATG-3 |  |
| *Lb6G30066*-S | 5-ATGGGTGGTGGTATGATGTAT-3 |  |
| *Lb6G30066*-A | 5-TCACTGCAGCGGACATAGACT-3 |  |
| *Lb8G36586*-S | 5-ATGTCCCACATCTCTGTTGAA-3 |  |
| *Lb8G36586*-A | 5-TTAGTTGGCTCCTCTTCGGTG-3 |  |
| *Lb7G34891*-OE-S | 5- CGGGGATCCTCTAGAGTCGACATGATGGATGCTCACTTGCCACA -3 |  |
| *Lb7G34891*-OE-A | 5- GCCCTTGCTCACCATGTCGACCTAGCAGTTGAGCGGATACCTCT -3 |  |
| *Lb5G28679*-OE-S | 5-CGGGGATCCTCTAGAGTCGACATGGAATCATCGAGTTTCCCTC-3 |  |
| *Lb5G28679*-OE-A | 5-GCCCTTGCTCACCATGTCGACTTAGTATGCCCCCGCGATCGGTA-3 |  |
| *Lb1G02274*-OE-S | 5-CGGGGATCCTCTAGAGTCGACATGGTCGTGGACGCTTACAGC-3 |  |
| *Lb1G02274*-OE-A | 5-GCCCTTGCTCACCATGTCGACCTATTCCGTAAACCCCGCTAAC-3 |  |
| *Lb1G07934*-OE-S | 5-CGGGGATCCTCTAGAGTCGACATGTTTGATCCTTTTCTGGGTGA-3 |  |
| *Lb1G07934*-OE-A | 5-GCCCTTGCTCACCATGTCGACCATCGGCGGTACACCAACC-3 |  |
| *Lb7G34782*-OE-S | 5-CGGGGATCCTCTAGAGTCGACATGGTGATTCTTATTACTCTGA-3 |  |
| *Lb7G34782*-OE-A | 5-GCCCTTGCTCACCATGTCGACCTACAAGAAGTGCAGCTCTGTC-3 | Construction of p35S:: GFP vectors digested with SalⅠ |
| *Lb2G14060*-OE-S | 5-CGGGGATCCTCTAGAGTCGACATGGCTAATTTCAATACGAACT-3 |  |
| *Lb2G14060*-OE-A | 5-GCCCTTGCTCACCATGTCGACTTACCTGCATCTCCCGCATGTGC-3 |  |
| *Lb3G16699*-OE-S | 5-CGGGGATCCTCTAGAGTCGACATGTCTACGGACCAGCTTACTC-3 |  |
| *Lb3G16699*-OE-A | 5-GCCCTTGCTCACCATGTCGACTCACATCTCCATCTTTAGACAA-3 |  |
| *Lb4G24818*-OE-S | 5-CGGGGATCCTCTAGAGTCGACATGGCTGTTCCGGCTGCCGGAA-3 |  |
| *Lb4G24818*-OE-A | 5-GCCCTTGCTCACCATGTCGACCTACAAGGAGTTGTTGGTATG-3 |  |
| *Lb6G30066*-OE-S | 5-CGGGGATCCTCTAGAGTCGACATGGGTGGTGGTATGATGTAT-3 |  |
| *Lb6G30066*-OE-A | 5-GCCCTTGCTCACCATGTCGACTCACTGCAGCGGACATAGACT-3 |  |
| *Lb8G36586*- OE-S | 5-CGGGGATCCTCTAGAGTCGACATGTCCCACATCTCTGTTGAA-3 |  |
| *Lb8G36586*- OE-A | 5-GCCCTTGCTCACCATGTCGACTTAGTTGGCTCCTCTTCGGTG-3 |  |
| *Lb1G07934-*primer1 | 5- ATATATGGTCTCGATTGCCGCCACCACCACCTCCTCGTT -3 | Construction of CRISPR knockout carrier |
| *Lb1G07934-*primer2 | 5- TGTAGAAGAGTACATAATAAGGTTTTAGAGCTAGAAATAGC -3 |  |
| *Lb1G07934-*primer3 | 5-AACCTTATTATGTACTCTTCTACAATCTCTTAGTCGACTCTAC-3 |  |
| *Lb1G07934-*primer4 | 5-ATTATTGGTCTCGAAACGAGGAGGTGGTGGTGGCGGC-3 |  |
