# Supplementary figures and images for: Genome-wide identification of bHLH transcription factors and functional analysis in salt gland development of the recretohalophyte sea lavender (Limonium bicolor)
Source: Hortic Res. 2024 Feb 2;11(4):uhae036. doi: 10.1093/hr/uhae036 (PMC11001596; doi:10.1093/hr/uhae036)

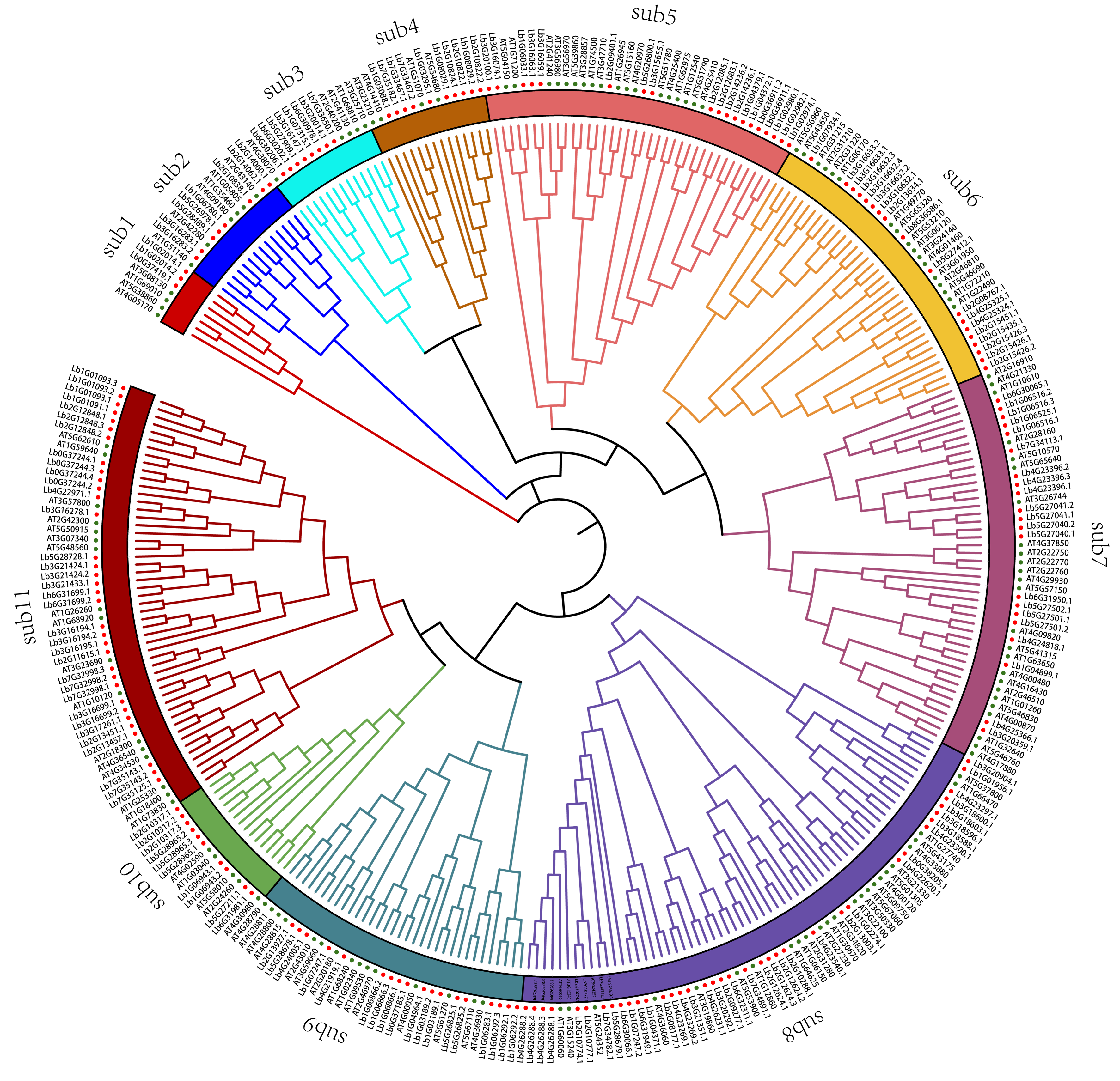

Supplement: Web_Material_uhae036 [file web_material_uhae036.zip › Figure S1.tif]

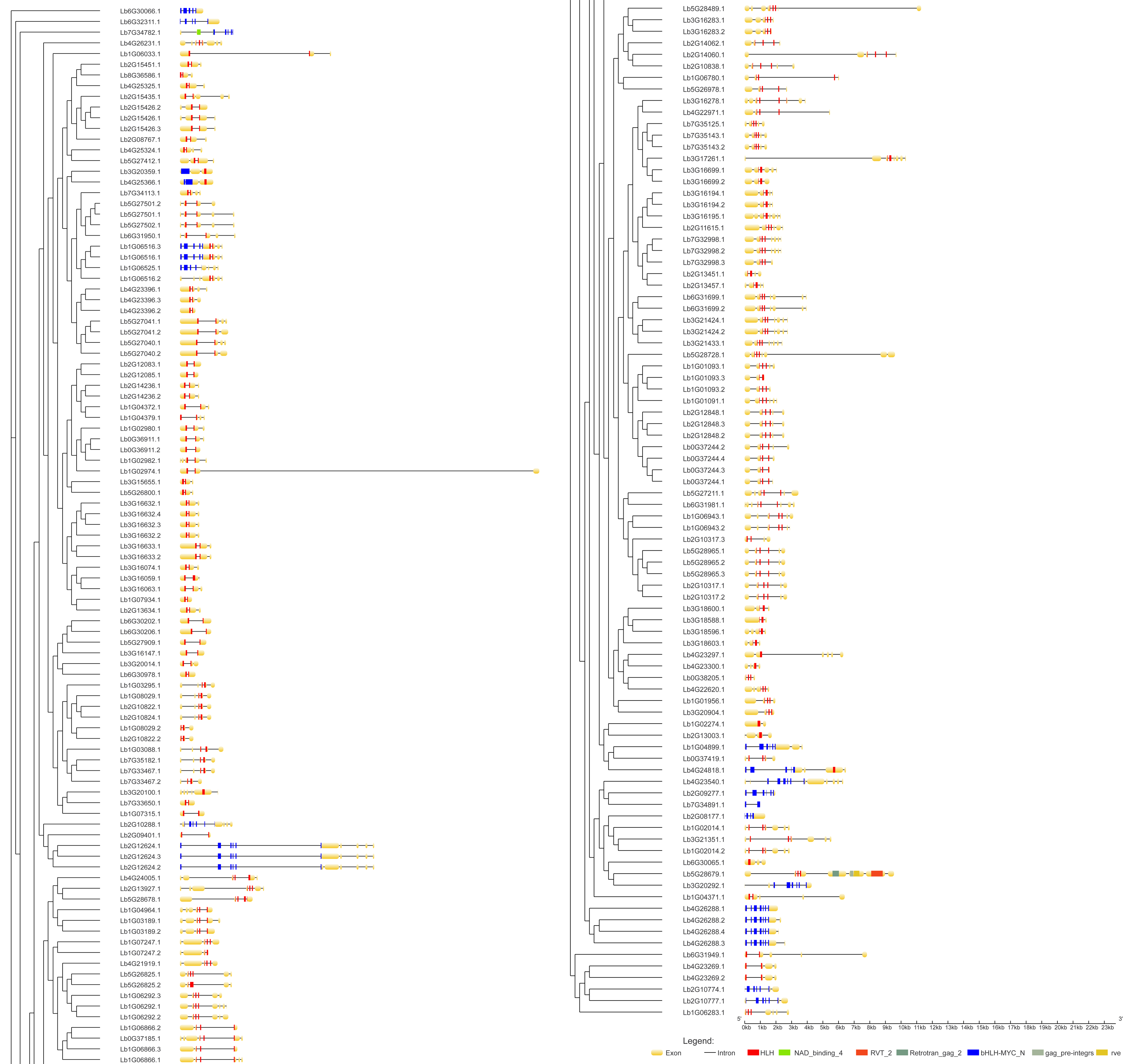

Supplement: Web_Material_uhae036 [file web_material_uhae036.zip › Figure S2.tif]

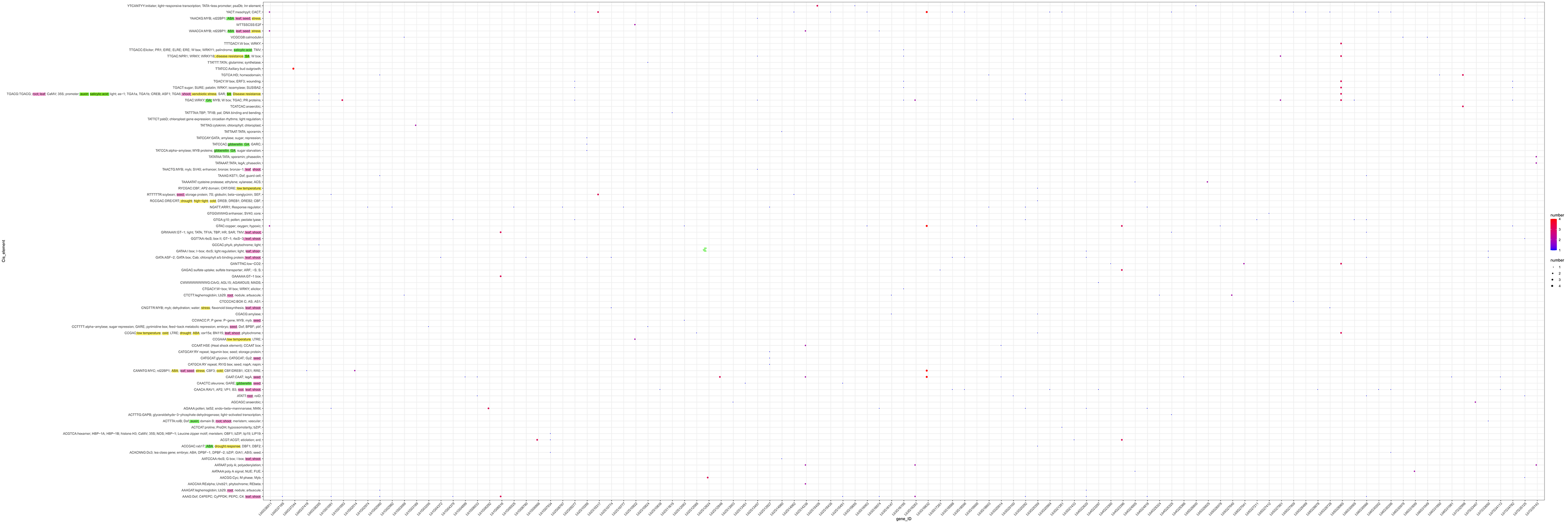

Supplement: Web_Material_uhae036 [file web_material_uhae036.zip › Figure S3.tif]

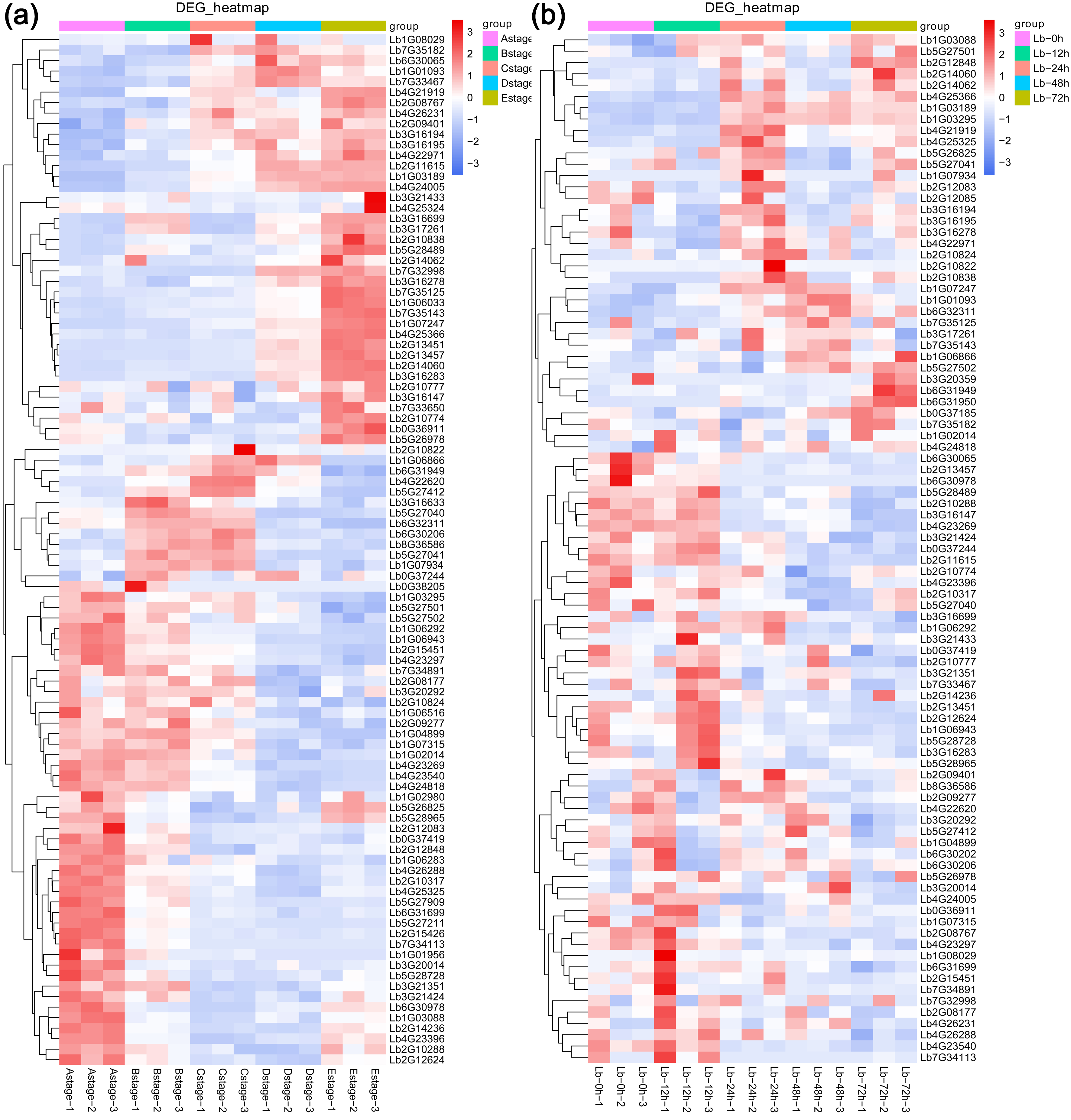

Supplement: Web_Material_uhae036 [file web_material_uhae036.zip › Figure S4.tif]

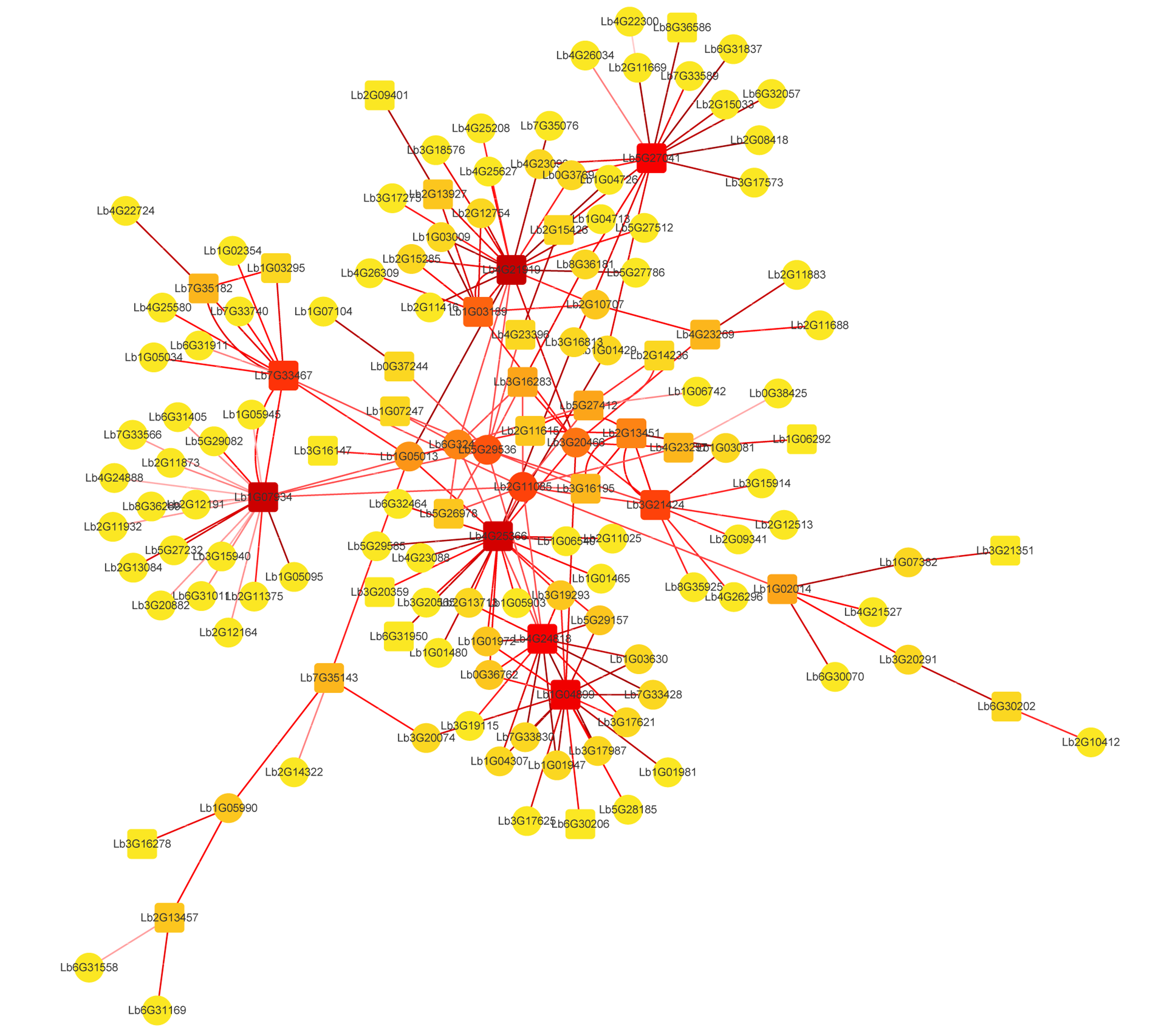

Supplement: Web_Material_uhae036 [file web_material_uhae036.zip › Figure S5.tif]
